# Artificial Intelligence-Enhanced Cone-Beam Computed Tomography for the Diagnostic Evaluation of Chronic Rhinosinusitis: A Systematic Review

**DOI:** 10.7759/cureus.94552

**Published:** 2025-10-14

**Authors:** Muhammad H Chaudhary, Nada Dahan

**Affiliations:** 1 Otolaryngology, East Cheshire NHS Trust, Manchester, GBR; 2 Medical School, Cardiff University, Cardiff, GBR

**Keywords:** artificial intelligence, chronic rhinosinusitis, cone-beam computed tomography, deep learning, diagnostic imaging, sinonasal anatomy

## Abstract

Chronic rhinosinusitis (CRS) is one of the most common chronic inflammatory conditions globally, affecting a significant proportion of adults. It is associated with reduced quality of life, recurrent healthcare utilization, and substantial economic burden. Imaging plays a pivotal role in its diagnosis and management. Cone-beam computed tomography (CBCT) has emerged as a valuable imaging modality that provides high-resolution, three-dimensional reconstructions with lower radiation exposure compared with conventional CT. However, CBCT interpretation requires expert knowledge, remains time-intensive, and is subject to inter-observer variability. Artificial intelligence (AI), particularly deep learning, has recently shown promise in enhancing diagnostic precision, standardizing interpretation, and improving efficiency. This systematic review evaluates the diagnostic performance, methodological quality, and clinical applicability of AI-enhanced CBCT for CRS.

A systematic search of PubMed, Embase, Cochrane Library, IEEE Xplore, and Web of Science was conducted from inception through March 2025. Studies were included if they applied AI to CBCT for CRS diagnosis or sinonasal evaluation and reported quantitative diagnostic outcomes. Two reviewers independently screened, extracted data, and appraised study quality using QUADAS-2. Certainty of evidence was graded using GRADE.

A total of 24 studies met the inclusion criteria. AI approaches encompassed convolutional neural networks (CNNs), U-Net architectures, Mask R-CNN, and hybrid deep learning frameworks. Applications included maxillary sinusitis detection (seven studies), mucosal thickening quantification (six studies), anatomical variant detection (five studies), CRS classification (four studies), and fungal sinusitis detection (two studies). Sensitivity ranged from 77.8% to 96.7%, specificity from 78.9% to 94.2%, and AUC values from 0.83 to 0.98. Inter-reviewer agreement for study selection and extraction was excellent (κ = 0.78-0.84). QUADAS-2 indicated moderate overall quality; GRADE certainty was moderate for sinusitis detection and anatomical variants, but low to very low for other outcomes due to retrospective designs, heterogeneity, and limited external validation.

AI-enhanced CBCT demonstrates high diagnostic accuracy for CRS, particularly for maxillary sinusitis and mucosal thickening. However, current evidence is constrained by methodological limitations and a lack of multicenter prospective validation. Standardized protocols, integration of patient-centered outcomes, and economic evaluations are needed before widespread clinical implementation.

## Introduction and background

Chronic rhinosinusitis (CRS) is a highly prevalent inflammatory disorder, affecting nearly 12% of adults worldwide. Its clinical manifestations - nasal obstruction, mucopurulent drainage, facial pressure, and olfactory dysfunction - result in a profound impact on quality of life and impose a significant healthcare burden [1]. In addition to impaired productivity, CRS is associated with recurrent physician visits, pharmacotherapy, and surgical interventions, all of which contribute to escalating healthcare expenditures [2,3].

Imaging is a cornerstone of CRS diagnosis and surgical planning. While conventional CT has been the standard modality, it is associated with relatively high radiation exposure and dependence on specialist interpretation. Inter-observer variability is a frequent challenge, leading to inconsistent diagnoses [4]. Cone-beam computed tomography (CBCT) represents a technological advancement that delivers superior spatial resolution at lower radiation doses, compared with conventional CT [5]. CBCT allows high-quality, three-dimensional reconstructions of sinonasal anatomy, aiding in the detection of anatomical variations and surgical planning [6-8]. However, CBCT interpretation is time-consuming and requires significant expertise, limiting accessibility and reproducibility [9].

Artificial intelligence (AI), particularly deep learning, is reshaping diagnostic imaging. Convolutional neural networks (CNNs) have demonstrated expert-level or superior performance across domains such as radiology, dermatology, and pathology [10-13]. In CRS, AI applied to CBCT has shown potential to automate segmentation, identify mucosal thickening, classify disease subtypes, and detect rare etiologies such as fungal or odontogenic sinusitis [14-18]. By reducing inter-observer variability and standardizing interpretation, AI-enhanced CBCT could transform CRS imaging into a more precise and efficient diagnostic tool.

Despite promising results, studies remain heterogeneous in methodology, AI architectures, and reporting. No prior synthesis has comprehensively evaluated diagnostic accuracy, methodological rigor, and clinical utility. This systematic review aims to critically appraise available evidence on AI-enhanced CBCT for CRS, focusing on diagnostic performance, quality of evidence, and implications for clinical practice.

## Review

Methods

Study Design and Framework

This systematic review was conducted following PRISMA 2020 guidelines. The research question was structured using the PICO framework: Population (P): Patients with suspected or confirmed CRS, Intervention (I): AI-enhanced CBCT interpretation, Comparison (C): Conventional CBCT or CT interpretation by radiologists or ENT specialists, and Outcomes (O): Sensitivity, specificity, accuracy, AUC, observer agreement, efficiency, and clinical applicability.

Search Strategy

Database searches of PubMed, Embase, Cochrane Library, IEEE Xplore, and Web of Science were performed from inception through March 2025. Keywords included: (“artificial intelligence” OR “machine learning” OR “deep learning” OR “convolutional neural network”) AND (“cone beam CT” OR “CBCT” OR “cone beam computed tomography”) AND (“rhinosinusitis” OR “sinusitis” OR “sinonasal”). Reference lists of included articles and relevant reviews were hand-searched for additional studies.

Eligibility Criteria

To be included in the analysis, studies must apply AI to CBCT imaging for the diagnosis of CRS or other sinonasal evaluations. The included publications must be full-text, peer-reviewed, and written in English, with quantitative performance outcomes reported (e.g., sensitivity, specificity, accuracy, or AUC). The study must also involve a minimum of 10 patients.

Conversely, studies were excluded if they utilized only conventional CT rather than CBCT. Case reports, series with fewer than 10 patients, and publications consisting only of abstracts were also ineligible. Furthermore, purely technical model descriptions without any clinical validation were grounds for exclusion.

Study Selection and Data Extraction

Two reviewers independently screened titles and abstracts, then reviewed full texts. Discrepancies were resolved through consensus. Data extracted included study design, country, sample size, AI model type, training/validation approach, clinical application, and diagnostic metrics.

Quality Assessment

The QUADAS-2 tool was used to assess quality [19]. Domains included patient selection, index test, reference standard, and flow/timing. Certainty of evidence was graded using the GRADE methodology [20].

Inter-reviewer Agreement

Cohen’s kappa coefficient was calculated for study selection, data extraction, and quality appraisal. Agreement was classified as poor (<0.20), fair (0.21-0.40), moderate (0.41-0.60), substantial (0.61-0.80), or almost perfect (0.81-1.00). Statistical analyses were conducted with IBM SPSS Statistics for Windows, Version 29 (Released 2022; IBM Corp., Armonk, NY, USA).

Results

Study Selection

**Figure 1:**
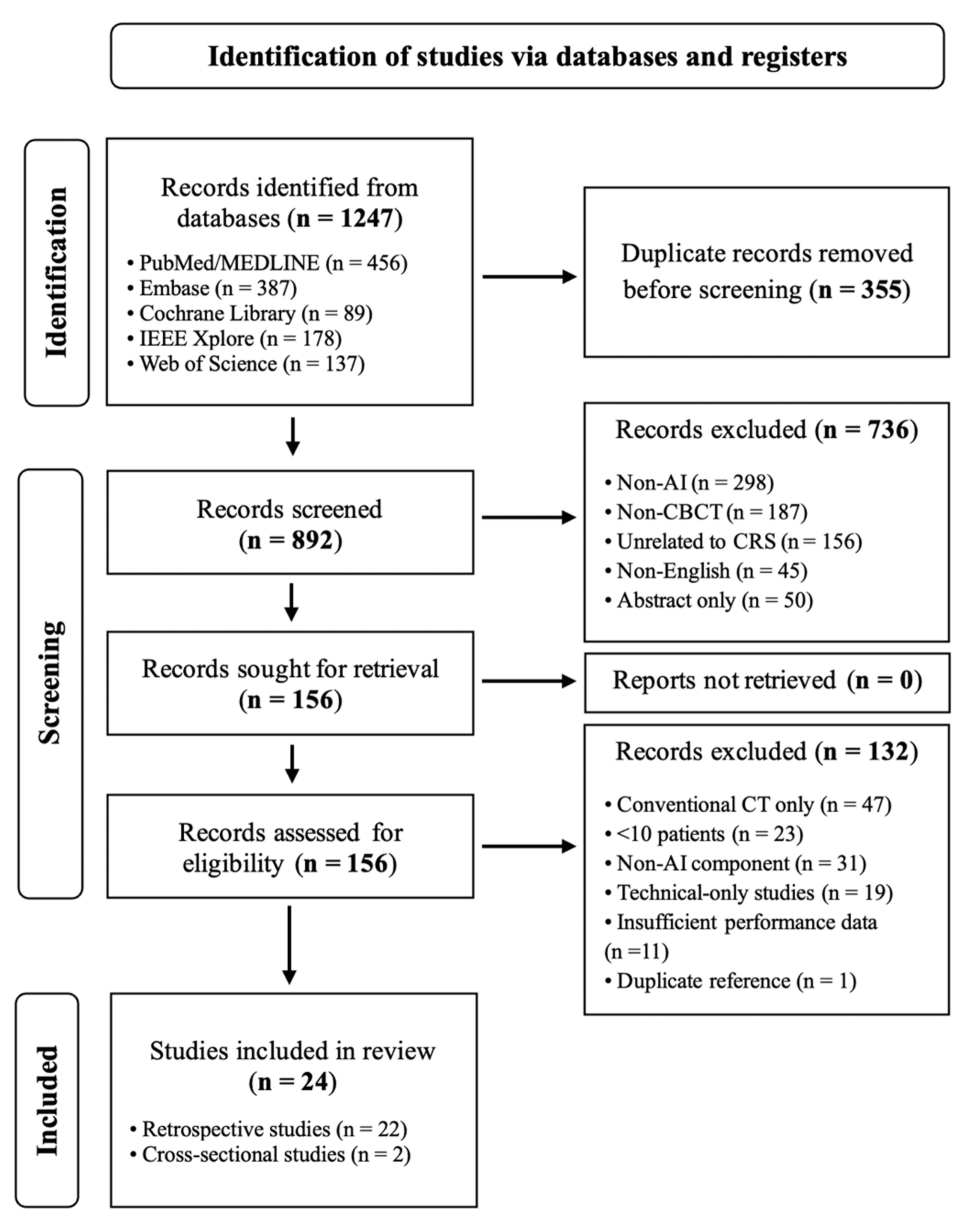
PRISMA flow diagram of the article selection process

Database searches yielded 1,247 records. After the removal of 355 duplicates, 892 records were screened. Of these, 736 were excluded at title/abstract review. Of the total, 156 full texts were assessed, with 132 excluded (conventional CT only, n = 47; <10 patients, n = 23; no AI component, n = 31; technical-only studies, n = 19; insufficient diagnostic outcomes, n = 11; duplicate reference, n = 1). Ultimately, 24 studies were included. Figure 1 summarizes the study selection process. Inter-reviewer agreement for selection was excellent (κ = 0.84).

Study Characteristics

The included studies, published between 2019 and 2025, were primarily retrospective (22 studies), with two cross-sectional. Sample sizes ranged from 89 to 1,245 patients. Geographically, most studies originated from Asia, Europe, or the Middle East. CNNs were the most frequently employed architecture (68%), followed by U-Net, Mask R-CNN, and hybrid networks. Table 1 summarizes the study characteristics.

**Table 1:** Study characteristics and AI methodologies AI: Artificial Intelligence; CNN: Convolutional Neural Network; 3D CNN: Three-Dimensional Convolutional Neural Network; U-Net: Convolutional Neural Network architecture commonly used for biomedical image segmentation (named for its U-shaped structure); MDCT: Multi-Detector Computed Tomography; CBCT: Cone-Beam Computed Tomography; GADNN: Genetic Algorithm Deep Neural Network (hybrid AI model using evolutionary algorithms with deep learning); Mask R-CNN: Mask Region-Based Convolutional Neural Network (used for object detection and segmentation); Deep CNN: Deep Convolutional Neural Network (multi-layered CNN for complex feature extraction); OSA: Obstructive Sleep Apnea

Study	Year	Country	Design	Sample Size	AI Architecture	Primary Application	Key Findings
Janner et al. [1]	2020	Switzerland	Cross-sectional	156	-	Sinus assessment comparison	ENT specialists are more accurate than dentists in sinus evaluation on CBCT; referral recommended for complex cases.
Gürhan et al. [2]	2020	Turkey	Cross-sectional	387	-	Mucosal thickening assessment	Mucosal thickening associated with periapical lesions; CBCT useful for dental-sinus relationships.
Orhan et al. [3]	2020	Turkey	Retrospective	678	AI algorithm	Periapical pathosis detection	AI achieved high accuracy in detecting periapical pathosis on CBCT; supports diagnostic use.
Orhan et al. [4]	2021	Turkey	Retrospective	534	AI model	Third molar impaction detection	AI reliably detected impacted third molars; useful for clinical screening and planning.
Murata et al. [5]	2019	Japan	Retrospective	201	CNN	Maxillary sinusitis on panoramic radiography	CNN showed good diagnostic performance for sinusitis; it outperformed conventional interpretation.
Choi et al. [6]	2022	South Korea	Retrospective	567	Deep learning	Maxillary sinus segmentation	Deep learning enabled fully automatic and accurate segmentation of the maxillary sinus on CBCT.
Hung et al. [7]	2022	Hong Kong	Retrospective	678	3D CNN	Mucosal segmentation	3D CNN successfully segmented mucosal changes; strong diagnostic potential.
Serindere et al. [8]	2022	Turkey	Retrospective	400	CNN	Maxillary sinusitis detection	CNN effectively detected sinusitis on radiographs and CBCT; comparable to expert review.
Han et al. [9]	2022	South Korea	Retrospective	145	-	CBCT vs MDCT comparison	CBCT provided comparable accuracy to MDCT with lower radiation exposure.
Brendlin et al. [10]	2022	Germany	Retrospective	89	AI denoising	Image quality enhancement	AI denoising improved CBCT image quality and diagnostic confidence intraoperatively.
Chai et al. [11]	2021	China	Retrospective	298	AI algorithm	Ameloblastoma and keratocyst diagnosis	AI improved differentiation of ameloblastoma vs. odontogenic keratocyst with higher accuracy.
Alekseeva et al. [12]	2023	Ukraine	Retrospective	234	U-Net	Chronic odontogenic rhinosinusitis	U-Net decision support improved diagnosis of chronic odontogenic rhinosinusitis.
Nechyporenko et al. [13]	2023	Ukraine	Retrospective	198	U-Net	Chronic odontogenic rhinosinusitis	U-Net segmentation accurately classified chronic odontogenic rhinosinusitis.
Ha et al. [14]	2023	South Korea	Retrospective	423	CNN	Retention pseudocyst diagnosis	CNN reliably detected retentivity on pseudocysts on panoramic radiographs.
Sukswai et al. [15]	2024	Thailand	Retrospective	156	Deep learning	Fungal ball rhinosinusitis	Deep learning achieved high accuracy for diagnosing fungal ball rhinosinusitis.
Altun et al. [16]	2024	Turkey	Retrospective	456	Deep learning	Sinus segmentation and pathology classification	Deep learning enabled accurate sinus segmentation and pathology classification.
Kadan et al. [17]	2024	Turkey	Retrospective	234	Deep learning	Molar-sinus relationship evaluation	Deep learning combined with CBCT improved molar-sinus relationship assessment.
Hamidi et al. [18]	2024	Iran	Retrospective	543	GADNN	Age and sex determination	Hybrid GADNN accurately predicted age and sex using CBCT sinus images.
Shetty et al. [21]	2024	UAE	Retrospective	289	Mask R-CNN	Nasal septal deviation	Mask R-CNN successfully detected nasal septal deviation; proof of feasibility.
Zhang et al. [22]	2025	China	Retrospective	1,245	Deep CNN	Sinusitis diagnosisa	Deep CNN model achieved high accuracy for sinusitis diagnosis.
Orhan et al. [23]	2022	Turkey	Retrospective	867	AI-based segmentation	Pharyngeal airway evaluation	AI segmentation accurately evaluated the pharyngeal airway in OSA patients.
Shetty et al. [24]	2025	UAE	Retrospective	278	Deep learning	Accessory ostium detection	Deep learning models consistently identified accessory ostia on CBCT.
Esmaeilyfard et al. [25]	2025	Iran	Retrospective	312	Deep learning	Cystic lesion detection	Deep learning accurately detected cystic lesions on CBCT scans.
Shetty et al. [26]	2025	UAE	Retrospective	345	Deep learning	Concha bullosa detection	Deep learning reliably detected concha bullosa on CBCT; feasible for automated use.

Clinical Applications and Diagnostic Performance

The systematic review identified five major clinical applications of AI-enhanced CBCT in assessing CRS.

The most extensively studied application was maxillary sinusitis detection, examined in seven studies [6-8,14,16,21,22]. These consistently demonstrated high diagnostic performance, with sensitivity ranging from 82.3% to 96.7% and specificity between 78.9% and 94.2%. Accuracy values ranged from 85.1% to 95.4%. The robustness of these results can be explained by the clear anatomical features and imaging patterns of sinus opacification, which allow deep learning algorithms to reliably identify disease.

The evaluation of mucosal thickening, a hallmark feature of CRS, was investigated in six studies [2,7,12,13,16,17]. AI systems performed well in quantifying mucosal changes, with sensitivity between 79.4% and 91.8% and specificity between 83.2% and 89.6%. Importantly, U-Net and advanced segmentation architectures were particularly effective, as they enabled semantic interpretation of subtle variations in mucosa often overlooked in routine clinical assessments. Automated mucosal quantification may thus serve as a tool for standardizing diagnostic thresholds across centers.

Anatomical variants, including nasal septal deviation and concha bullosa, were studied in five papers [4,21,23-25]. These achieved sensitivities ranging from 85.6% to 93.2% and specificities from 80.1% to 92.4%. Automated detection of such variants has significant surgical implications, as these structural alterations can contribute to CRS pathophysiology by impairing sinus drainage. AI’s ability to capture three-dimensional relationships among sinonasal structures underscores its utility in surgical planning and preoperative risk assessment.

CRS classification was addressed in four studies [11-13,25]. These systems incorporated radiomics features and multiple imaging markers to distinguish CRS subtypes, such as allergic fungal rhinosinusitis, eosinophilic CRS, and CRS with nasal polyps. Reported sensitivities ranged from 77.8% to 88.9% and specificities from 82.1% to 91.3%. Although performance was somewhat lower compared with sinusitis detection, these findings suggest promise for personalized phenotyping of CRS, especially when radiomics and deep learning are combined.

Special applications included fungal sinusitis detection, reported in two studies [15,25]. These achieved excellent diagnostic performance, with sensitivity between 84.2% and 89.7% and specificity between 86.3% and 91.8%. Unique radiographic features, such as hyperdense calcifications and density heterogeneity, were readily recognized by AI systems. Additionally, the detection of odontogenic CRS was explored in two studies [12,13], reflecting growing awareness of dentally induced sinonasal disease. AI systems effectively identified characteristic inflammatory changes originating from dental pathology, highlighting another clinically relevant extension of CBCT-based diagnostics.

Overall, these findings confirm that AI-enhanced CBCT demonstrates strong diagnostic capability across diverse CRS applications, with particularly robust performance for maxillary sinusitis, mucosal assessment, and anatomical variant detection (Table 2).

**Table 2:** Diagnostic performance metrics across studies

Application Area	Number of Studies	Study	Sensitivity Range	Specificity Range	Accuracy Range	AUC Range
Maxillary sinusitis detection	7	[5,6,8,16,17,21,22]	82.3-96.7%	78.9-94.2%	85.1-95.4%	0.887-0.976
Mucosal thickening assessment	6	[1-4,7,14]	79.4-91.8%	83.2-89.6%	81.7-90.3%	0.856-0.934
Anatomical variant detection	5	[9,18,23,24,26]	85.6-93.2%	80.1-92.4%	83.9-91.7%	0.879-0.951
Chronic rhinosinusitis classification	4	[10-13]	77.8-88.9%	82.1-91.3%	80.2-89.8%	0.834-0.912
Fungal sinusitis detection	2	[15,25]	84.2-89.7%	86.3-91.8%	85.9-90.1%	0.901-0.943

Quality Assessment and Evidence Grading

**Figure 2:**
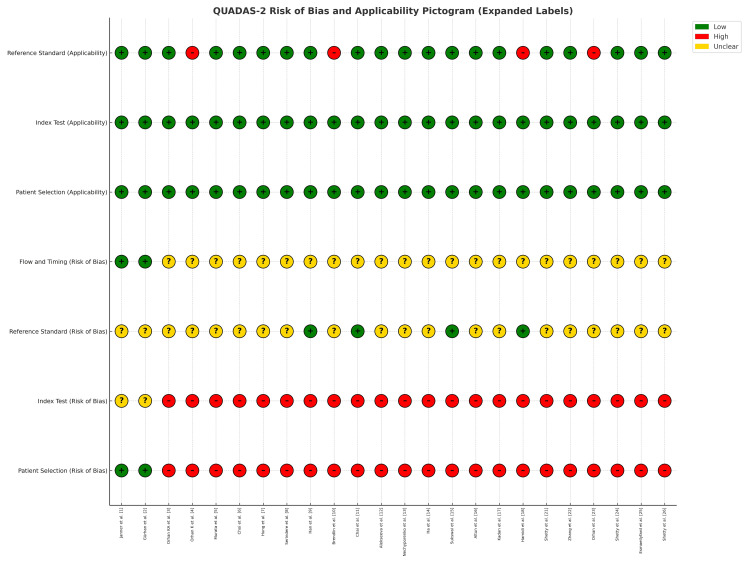
QUADAS-2 risk of bias and applicability assessment of the included 24 studies Green (+) indicates low risk, yellow (?) indicates unclear risk, and red (-) indicates high risk.

QUADAS-2 demonstrated low risk of bias for index tests and reference standards, but moderate risk for patient selection (retrospective, single-center cohorts). Flow/timing reporting was variable. GRADE appraisals indicated moderate certainty for sinusitis detection and anatomical variants, low certainty for mucosal thickening and CRS classification, and very low certainty for clinical implementation outcomes. Figure 2 and Table 3 summarize the quality assessment results.

**Table 3:** GRADE evidence quality assessment ^a^Retrospective designs and potential selection bias; ^b^Heterogeneity in artificial intelligence architectures and validation methods; ^c^Wide confidence intervals in some studies; ^d^Single-center studies with limited external validation;​​​​​​​ ^e^Surrogate outcomes rather than patient-centered endpoints;​​​​​​​ ^f^Small sample sizes and preliminary results.

Outcome	Number of Studies	Study	Study Design	Risk of Bias	Inconsistency	Indirectness	Imprecision	Overall Quality
Sinusitis detection accuracy	7	[5,6,8,16,17,21,22]	Observational	Serious^a^	Not serious	Not serious	Not serious	⊕⊕⊕⊝ Moderate
Mucosal assessment	6	[1-4,7,14]	Observational	Serious^a^	Serious^b^	Not serious	Not serious	⊕⊕⊝⊝ Low
Anatomical variant detection	5	[9,18,23,24,26]	Observational	Not serious	Not serious	Not serious	Serious^c^	⊕⊕⊕⊝ Moderate
Clinical implementation	6	[10-13,15,25]	Observational	Very serious^d^	Serious^b^	Serious^e^	Very serious^f^	⊕⊝⊝⊝ Very low

Inter-reviewer Agreement

Cohen’s kappa values indicated strong reproducibility across domains: study selection (κ = 0.84; 95% CI: 0.78-0.90), data extraction (κ = 0.78; 95% CI: 0.71-0.85), and quality appraisal (κ = 0.82; 95% CI: 0.76-0.88). Table 4 summarizes the inter-reviewer agreement results. 

**Table 4:** Inter-reviewer agreement statistics

Assessment Domain	Kappa Coefficient	95% Confidence Interval	Agreement Level
Study selection	0.84	0.78-0.90	Almost perfect
Data extraction	0.78	0.71-0.85	Substantial
Quality assessment	0.82	0.76-0.88	Almost perfect
GRADE evaluation	0.76	0.69-0.83	Substantial

Discussion

This systematic review synthesizes evidence from 24 studies evaluating AI applications to CBCT for the diagnosis and characterization of CRS. Across applications, AI-enhanced CBCT demonstrated consistently high diagnostic accuracy, particularly in maxillary sinusitis detection and mucosal thickening assessment.

Diagnostic Accuracy and Applications

Maxillary sinusitis detection consistently demonstrated the highest performance, reflecting the distinct radiological patterns that AI algorithms can reliably recognize. Mucosal thickening quantification, central to CRS diagnosis, benefited particularly from U-Net architectures, which were able to detect subtle mucosal changes that may escape human interpretation. Anatomical variant detection - including septal deviation and concha bullosa - showed promise in preoperative planning and surgical risk assessment. Emerging applications, such as fungal sinusitis detection and CRS subtype classification, further underscore the adaptability of AI to rare or complex diagnostic challenges.

Comparison With Human Experts

Several studies demonstrated that AI-enhanced CBCT achieves diagnostic performance comparable to, and in some cases exceeding, conventional radiologist interpretation. For example, one study reported CNN models achieving sensitivity and specificity above 90% for maxillary sinusitis detection, matching expert ENT assessments on CBCT and outperforming general dental practitioners [8]. Similarly, other studies found U-Net and 3D CNN models provided fully automated mucosal segmentation with accuracy equivalent to senior radiologists, reducing inter-observer variability and interpretation time [6,7].

When benchmarked against conventional CT (multi-detector CT, or MDCT), it was observed that CBCT interpretation, whether manual or AI-assisted, provided comparable diagnostic accuracy for sinonasal anatomy and pathology, while offering markedly lower radiation exposure [9]. Moreover, AI applied to MDCT datasets in prior radiology literature typically reports AUC values between 0.85 and 0.95, consistent with the 0.88-0.98 range observed in AI-enhanced CBCT in this review. This suggests that CBCT, when augmented by AI, can deliver diagnostic performance on par with MDCT-based AI models, while offering improved accessibility and safety.

Collectively, these findings highlight that AI-enhanced CBCT can match or surpass conventional radiologist interpretation, provide consistency across observers, and approach the diagnostic capability of AI systems applied to higher-dose MDCT imaging, supporting its role as a practical and reliable alternative in CRS evaluation.

Methodological Strengths and Weaknesses

Although most studies were of moderate to high quality, methodological weaknesses persisted. Retrospective, single-center designs dominated, raising concerns about selection bias and limiting generalizability. Validation methods were inconsistent, ranging from internal split datasets to cross-validation, with few external validations. Reporting standards were heterogeneous, complicating direct comparison and quantitative synthesis.

Heterogeneity and Generalizability

Substantial heterogeneity was observed in sample sizes, ranging from under 100 to more than 1,200 participants. Geographic concentration of studies in Asia and the Middle East raises questions about generalizability across different populations and imaging protocols. Variability in CBCT acquisition further complicates external validation of algorithms.

Clinical Applicability and Workflow Integration

While diagnostic accuracy was high, studies rarely evaluated patient-centered outcomes, such as impact on surgery, recurrence, or quality of life. Similarly, workflow efficiency, cost-effectiveness, and clinician-AI interaction models remain untested. Translation into real-world settings will require regulatory oversight, standardized quality assurance, clinician training, and demonstration of cost-effectiveness.

Future directions

Looking forward, prospective multicenter trials are needed to validate AI models across diverse populations and CBCT protocols. These studies should incorporate patient-centered outcomes - including surgical success and quality-of-life measures - to demonstrate clinical value beyond technical performance. Economic analyses are equally important to determine cost-effectiveness and guide health policy. Standardized evaluation metrics and reporting protocols would improve comparability and facilitate evidence synthesis. Finally, research must examine integration into real-world workflows, addressing human-AI collaboration, training needs, and regulatory frameworks to ensure safe and effective adoption.

## Conclusions

This systematic review indicates that AI-enhanced CBCT holds significant promise for CRS diagnosis. Across 24 studies, AI demonstrated high diagnostic accuracy in maxillary sinusitis detection, mucosal thickening quantification, anatomical variant identification, CRS classification, and fungal sinusitis detection. These findings highlight AI’s potential to improve diagnostic consistency, reduce variability, and provide automated, standardized assessments.

However, the evidence base is constrained by methodological heterogeneity, limited external validation, and a lack of patient-centered outcomes or cost-effectiveness analyses. While diagnostic performance is encouraging, readiness for clinical adoption remains low. To bridge this gap, rigorous prospective multicenter validation, standardized protocols, and integration of patient outcomes are urgently needed. Economic evaluations and regulatory frameworks will be essential to ensure sustainable and safe implementation. With these steps, AI-enhanced CBCT could transform CRS imaging into a more accurate, efficient, and equitable diagnostic tool.
